# TRPC1/5-Ca_*V*_3 Complex Mediates Leptin-Induced Excitability in Hypothalamic Neurons

**DOI:** 10.3389/fnins.2021.679078

**Published:** 2021-06-11

**Authors:** Paula P. Perissinotti, Elizabeth Martínez-Hernández, Erika S. Piedras-Rentería

**Affiliations:** Cell and Molecular Physiology Department and Neuroscience Division of the Cardiovascular Research Institute, Loyola University Chicago, Maywood, IL, United States

**Keywords:** Ca_*V*_3.1, Ca_*V*_3.2, TRPC channel, hypothalamus, leptin, POMC

## Abstract

Leptin regulates hypothalamic POMC^+^ (pro-opiomelanocortin) neurons by inducing TRPC (Transient Receptor Potential Cation) channel-mediate membrane depolarization. The role of TRPC channels in POMC neuron excitability is clearly established; however, it remains unknown whether their activity alone is sufficient to trigger excitability. Here we show that the right-shift voltage induced by the leptin-induced TRPC channel-mediated depolarization of the resting membrane potential brings T-type channels into the active window current range, resulting in an increase of the steady state T-type calcium current from 40 to 70% resulting in increased intrinsic excitability of POMC neurons. We assessed the role and timing of T-type channels on excitability and leptin-induced depolarization *in vitro* in cultured mouse POMC neurons. The involvement of TRPC channels in the leptin-induced excitability of POMC neurons was corroborated by using the TRPC channel inhibitor 2APB, which precluded the effect of leptin. We demonstrate T-type currents are indispensable for both processes, as treatment with NNC-55-0396 prevented the membrane depolarization and rheobase changes induced by leptin. Furthermore, co-immunoprecipitation experiments suggest that TRPC1/5 channels and Ca_*V*_3.1 and Ca_*V*_3.2 channels co-exist in complex. The functional relevance of this complex was corroborated using intracellular Ca^2+^ chelators; intracellular BAPTA (but not EGTA) application was sufficient to preclude POMC neuron excitability. However, leptin-induced depolarization still occurred in the presence of either BAPTA or EGTA suggesting that the calcium entry necessary to self-activate the TRPC1/5 complex is not blocked by the presence of BAPTA in hypothalamic neurons. Our study establishes T-type channels as integral part of the signaling cascade induced by leptin, modulating POMC neuron excitability. Leptin activation of TRPC channels existing in a macromolecular complex with T-type channels recruits the latter by locally induced membrane depolarization, further depolarizing POMC neurons, triggering action potentials and excitability.

## Introduction

Leptin regulates energy homeostasis and serves as a satiety afferent signal in the homeostatic control of adipose tissue mass (Schwartz et al., 2000; Harvey and Ashford, 2003), reducing food intake, increasing energy expenditure and regulating the reward value of nutrient (Ahima and Flier, 2000; Domingos et al., 2011; Williams and Elmquist, 2012). Leptin exerts its physiological action through its specific receptor (LRb), which is highly expressed in hypothalamus and other brain areas (Shioda et al., 1998). Leptin’s effects on hypothalamic homeostatic feeding circuits are well established (Kenny, 2011); it negatively modulates orexigenic agouti-related peptide (AgRP)/neuropeptide Y (NPY) neurons by Kv2.1 channel-mediated membrane hyperpolarization (Baver et al., 2014). In contrast, anorexigenic POMC positive neurons are depolarized by leptin (Cowley et al., 2001). This depolarization is mediated via a Jak2-PI3 kinase-PLCγ pathway that ultimately activates TRPC channel activity (Qiu et al., 2010). Similarly, a subset of POMC neurons in the arcuate nucleus responsive to serotonin *via* the 5-HT_2C_ receptor are also activated *via* TRPC channels, suggesting TRPC channels are a common signaling mechanism mediating anorexigenic signaling in the hypothalamus (Sohn et al., 2011). TRPC5 channels are also the molecular mediators of the acute leptin and serotonin effect in POMC neurons (Gao et al., 2017).

The role of TRPC channels in POMC neuron excitability is clearly established; however, it is not known whether their activity alone is sufficient to trigger excitability. Here we used cultured hypothalamic neurons from mice to characterize the role of T-type Ca^2+^ channels and leptin-induced POMC neuron excitability. Our data demonstrate T-type channels are necessary for POMC neuron excitability, by being involved in the excitatory cascade induced by leptin in these neurons. Blockade of either TRPC or T-type channel function prevents the effect of leptin on hypothalamic neuron excitability. Moreover, we demonstrate that: (a) TRPC1 and TRPC5 channels co-immunoprecipitate with T-type channels in the hypothalamus, (b) TRPC-T-type channel complexes exist in a functional microdomain, and (c) TRPC-induced depolarization in these domains triggers neuronal excitability *via* recruitment of T-type channels. Thus, this study confirms T-type channels constitute a target to modulate leptin-activated neurons and their functions, such as energy balance and food intake.

## Materials and Methods

The animal protocols used in this study were reviewed and approved by an independent Institutional Animal Care and Use Committee (IACUC 2016032). Mixed background (129S1/Sv-Oca2 + Tyr + Kitl + C57BL/6) WT and EGFP-POMC^+^ mice [129S1/Sv-Oca2 + Tyr + Kitl + C57BL/6-Tg(Pomc-EGFP)1Low/J] (The Jackson Laboratory, RRID:IMSR_JAX:009593) were fed on an *ad libitum* standard commercial pellet diet. No exclusion criteria were pre-determined. Altogether, 12 newborn pups were used for hypothalamic cultures (1 pup per cell culture) and 17 adult mice (male) for immunoprecipitation experiments. The study was not pre-registered. Experiments were conducted in the afternoon.

### Neuron Cultures

Whole hypothalamus were dissected from newborn mice (postnatal day 0, P0) and cultured as described in Perissinotti et al. (2014). Cells plated at a density of 25,000−35,000/coverslip and kept in a 5% CO_2_ humidified atmosphere at 37°C. Newborn pups (no sex determination) were killed by decapitation after cold-induced anesthesia, and their brains rapidly removed prior to hypothalamus dissection; each culture was generated from two independent mice, each plated on 6 coverslips (electrophysiology and ICC data was generated from at least three independent batches of cultures, N = 6 mice). Cryo-anesthesia abolished perception of pain of pups. This method has been validated by Veterinary Services to minimize animal suffering.

### Immunocytochemistry (ICC)

WT neurons at 8−11 DIV were prepared as described (Perissinotti et al., 2014). Primary antibody dilutions were: POMC 1:200 (Novus, RRID:AB_791643); AgRP, 1:50 (Sta. Cruz, RRID:AB_2258141), Ca_*V*_3.1 1:200 (Alomone, RRID:AB_2039779) and 1:50 (Millipore, Cat.# MABN464), Ca_*V*_3.2 1:5 (supernatant, NeuroMab, RRID:AB_2069551) and 1:200 (Sta. Cruz, RRID:AB_2259537); TRPC1 (RRID:AB_2040234) 1:100 and TRPC5 (RRID:AB_2040241) 1:120 (Alomone). Secondary antibodies: Alexa-488 Goat anti-mouse (RRID:AB_2633275) and goat anti-rabbit (RRID:AB_143165), −594 goat anti-rabbit (RRID:AB_2762824), and −647 goat anti-chicken (RRID:AB_2762845), 1:2,000 (Molecular Probes, Eugene, OR). Image acquisition was done using a Olympus IX80 microscope, analyzed by deconvolution and processed with ImageJ freeware (NIH) (Schneider et al., 2012).

### Immunoprecipitation (IP)

Adult mice (24−30 g) were deeply anesthetized with 2% Isofluorane, then quickly decapitated; whole brains or hypothalamus were isolated according to guidelines of the IACUC. At least two whole brains or two whole hypothalami were needed per n to assess the presence of complexes with Ca_*V*_3.1 (α_1G_) or TRPC1, whereas only one whole brain/one hypothalamus were needed per n for Ca_*V*_3.2 (α_1H_) or TRPC5 complexes; each experiment was replicated three times. Tissues were homogenized using a Bullet blender tissue homogenizer using 0.5 mm zirconium oxide beads (Next Advance) and spun down at 1,300 × g to eliminate debris. A fraction of the supernatant was reserved before immunoprecipitation and stored at −80 C until processing (input); the remaining volume was divided up in equal parts for all experiments. Samples were then processed by addition of the primary antibody and incubation for 1 h at 4°C (antibodies: α1H (Santa Cruz Biotechnology, Inc., Santa Cruz, CA, United States, RRID:AB_2259537), α1G [Millipore, Cat. #MABN464); TRPC5 (Alomone, RRID:AB_2040241)], TRPC1 (Santa Cruz Biotechnology, Inc., Santa Cruz, CA, United States, RRID:AB_2207905), and IgG (Cat. #20008−1−100 and #20009−1−100, Alpha Diagnostics, San Antonio, TX) and overnight incubation, followed by incubation for 1 h with protein A/G agarose beads (Biovision, Mountain View, CA) on a shaking plate at 4°C. The samples were washed and then precipitated in 0.1 M glycine pH 3.5 and neutralized with 0.5 M Tris⋅HCl and 1.5 M NaCl pH 7.4 before SDS-PAGE electrophoresis (8%, at 100 V for 90 min) followed by transfer to a PVDF membrane (BioRad). Membranes were washed in Tris-buffered saline (TBS) + Tween (TBST; 0.05% Tween 20), blocked for 1 h in TBST + 5% milk at room temperature, and incubated at 4°C overnight with α1H polyclonal (1:2,000), α1G monoclonal (1:500), TRPC1 monoclonal (1:1,000), or TRPC5 polyclonal (1:1,000) antibody. Incubation with goat anti-rabbit (RRID:AB_1185567) horseradish peroxidase (HRP)- or anti-mouse (RRID:AB_228307) HRP-conjugated secondary antibodies was done at room temperature (1:2,000; Pierce). Blots were exposed to developing agent (Supersignal Femto Dura, Pierce) before analysis with a ChemiDoc MP System (BioRad).

### Electrophysiology

Whole-cell patch clamp recordings were performed from cultured hypothalamic neurons from 8 to 11 DIV using an Axopatch 200B amplifier (Axon instruments, Union City, CA) at room temperature. Data were acquired at 1 kHz and digitized at 20 kHz using a Digidata 1322A analog-to-digital converter. Pipettes pulled from borosilicate glass (Warner Instruments, Hamden, CT) had resistances of 3.5–4.5 MΩ when filled with intracellular solutions. Cells with series resistance (*R*_*s*_) < 20 MΩ were used; *R*_*s*_ was compensated online (>80%). Data was acquired with and analyzed with pClamp 10 software (Molecular Devices). Cell capacitance was measured from a transient current evoked by a 5 mV depolarizing step from a holding potential of -90 mV.

Calcium currents were recorded in an external solution containing (in mM) 5 CaCl_2_, 140 TEA-Cl, 10 HEPES and 10 glucose (pH 7.4, 300 mOsmol/kgH_2_O), using an intracellular solution containing (in mM) 108 CsMeSO_3_, 4 MgCl_2_, 10 Cs-EGTA, 9 HEPES, 5 ATP-Mg, 1 GTPLi and 15 phosphocreatine-Tris (pH 7.4, 290 mOsmol/kgH_2_O). Voltage control was improved by increasing cell impedance using extracellular TEA and intracellular cesium to block K^+^ conductances. I–V curve properties such as its negative slope and reversal potential were monitored for appropriate voltage control. For the study of calcium current properties, we avoided recording from neurons older than 10 DIV because the possibility of space-clamp problems.

Resting membrane potential (RMP) and APs were recorded in external solution containing (in mM) 135 NaCl, 5 KCl, 2 CaCl_2_, 1 MgCl_2_, 10 HEPES, 10 glucose (pH 7.4, 300 mosmol/kgH_2_O) and intracellular solution containing (in mM) 110 K-gluconate, 20 KCl, 2 MgCl_2_, 1 EGTA, 10 HEPES, 2 ATP-Mg, 0.25 GTP-Li and 10 phosphocreatine-Tris (pH 7.4, 290 mosmol/kgH_2_O). Drugs: NNC-550396 dihydrochloride (Cat. #2268) and Leptin (Cat. #2985) were purchased from Tocris (Bristol, United Kingdom).

Square protocols to obtain I–V curves and T-type steady-state activation and inactivation were done as described in Bean (1985) and Perissinotti et al. (2015).

#### Current-Voltage Relationships (I–V Curves)

Currents were elicited from a holding potential (V_*H*_) = −90 mV and depolarized for 150 ms to a test potential (V_*T*_) = −70 to + 60 mV, in 10 mV increments.

#### T-type Current—Rate of Membrane Potential Depolarization Relationship

Different rates of membrane potential depolarization preceding the first action potential were obtained from hypothalamic neurons by changing the rate of current ramp stimulations (20−100 pA/s) in current clamp configuration: 40, 90, 180, 290, or 360 mV/s (V_*H*_ = −75 mV). These voltage templates were used in voltage clamp configuration to stimulate calcium currents. The membrane potential was held at a V_*H*_ of −50 or −90 mV before clamping the voltage at −75 mV for 10 ms to run the stimulation protocol. The low-voltage activated (LVA) current component contribution was determined by the subtraction method (Bean, 1985): High-voltage activated (HVA) currents obtained at V_*H*_ = −50 mV were subtracted from those obtained at V_*H*_ = −90 mV (HVA plus LVA currents).

#### Steady-State Analysis

Steady-state activation (SSA) was analyzed with protocols stepping from V_*H*_ = −90 (or −50) mV to V_*T*_ = −90 to 0 mV (ΔV = 10 mV) for 12 ms followed by repolarization to −100 mV to evoke inward tail currents. Data were fitted by a single Boltzmann function of the form *I*_*max*_/[1 + exp ^(^*^*V*^_50_^– V^*^)/^*^*k*^*] + *m*, were *I*_*max*_ is maximal current, *V*_50_ is half-voltage of activation, *k* is slope factor, and *m* is baseline. Steady-state inactivation (SSI) was determined by stepping the membrane potential to various pre-pulse voltage levels (V_*pre*_ = −110 to 0 mV, ΔV = 10 mV) for 1 s before depolarization to a fixed test level (−30 mV) to evoke channel opening. The resulting data were also fitted to a Boltzmann function.

Steady-state current (I_*stst*_) was calculated from the formula I = G^∗^ (V- E_*Nerns*__*t(*__*ion*__)_), where the channel conductance G is multiplied by the channel’s open probability (I/I_*max*_, SSA) and availability (I/I_*max*_, SSI), which were obtained experimentally from the T-type steady state activation (SSA) or inactivation (SSI) curves adjusted to a Boltzmann equation (Bijlenga et al., 2000; Lambert et al., 2014; Rivero-Echeto et al., 2020). Specifically, steady-state current (I_*stst*_) was calculated with the steady stated formula Iss (V_*T*_) = G ^∗^ I/I_*max, SSA*_ (V_*T*_) ^∗^ I/I_*max, SSI*_ (V_*T*_)^∗^(V_*T*_- 50 mV), where V_*T*_ is the test voltage, G is the channel conductance (arbitrarily set at 1), I/I_*max,SSA*_ is the fraction of T-type channels activated at V_*T*_, I/I_*max,SSI*_ is the fraction of T-type channels available at V_*T*_ and 50 mV is the experimentally equilibrium potential for calcium.

#### Resting Membrane Potential

RMP was recorded in continuous trace mode without current injection for 20 s, and averaged; voltages were corrected for liquid junction potentials.

#### Input Resistance

Whole cell input resistance (*R*_*in*_) was determined in response to current steps (−80 to 20 pA, stepping each 20 pA). A holding current was applied to set the membrane potential at−60 mV.

#### Membrane Excitability

AP discharges were triggered by consecutive depolarizing ramps at 20, 40, and 60 pA/s rates for 1.5 s (Balasubramanyan et al., 2006; Lu et al., 2006). A holding current was applied to set the membrane potential at −75 mV. The rheobase was determined as the minimum amount of current required for firing an action potential.

### Statistical Analysis

These experiments were exploratory with no pre-determined end-point and no data was excluded from the analyses. No blinding, no randomization, and no sample size calculation were performed. All measurements were done in at least three independent cell cultures. Results are presented as mean ± SEM. The parameter “n” indicates number of cells unless otherwise stated and “N” the number of animals. No test for outliers has been applied. The normality of data distribution was assessed by the Kolmogorov-Smirnov test. Statistical analysis was performed with the Sigma Plot 11 Software. Statistical significance (*P* < 0.05) was determined using either one-way analysis of variance (ANOVA) with Fisher LSD’s *post hoc* test for comparisons between multiple groups or an independent-samples *t*-test for comparisons between two groups. Statistical significance (*P* < 0.05) for two proportions was determined using the *Z*-test.

## Results

**FIGURE 1:**
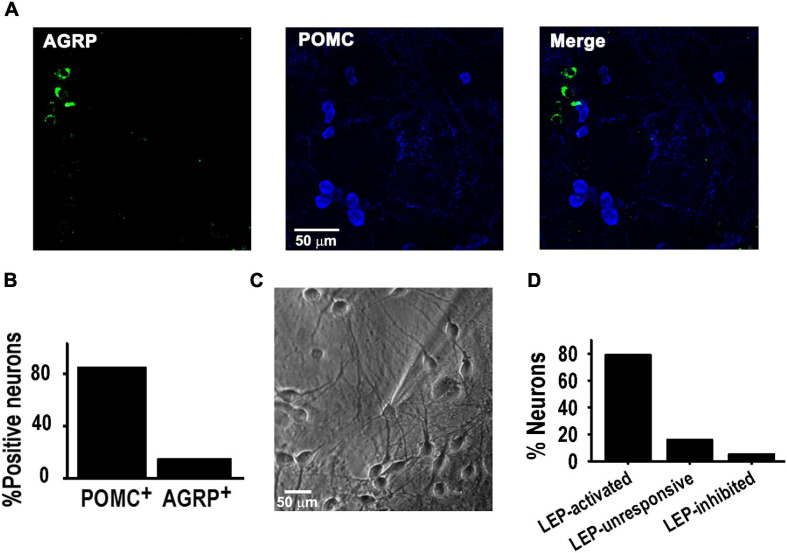
POMC neurons activated by leptin in culture. **(A)** Example of confocal images showing neurons positive for AGRP (green) or POMC (blue) antibodies in hypothalamus culture at 8–10 DIV. **(B)** Percentage of neurons positive for AGRP or POMC antibodies (*n* = 182). **(C)** Microscopic image of cultured hypothalamic neuron at 8 DIV. **(D)** Percentage of WT neurons activated or inhibited by 100 nM leptin (*n* = 19), using a 20 pA/s depolarizing ramp stimulus.

Hypothalamic cultures were studied from 8 to 11 days *in vitro* (DIV). Immunocytochemistry analysis (ICC) showed that POMC and NPY/*AGRP* neurons were present in the culture, with POMC^+^ neurons (POMC) being the majority (85 vs. 15%, *P* < 0.05, *Z*-test) (Figures 1A,B); note that Figure 1A figure does not display an average distribution of POMC^+^ and AGRP^+^ positive neurons, as it was difficult to find such ratios within the same area during confocal image acquisition. Electrophysiological experiments confirmed 15% of all studied neurons where neither activated nor inhibited by leptin; whereas 80% of all neurons were leptin-activated (Figures 1C,D), consistent with the ICC data. In contrast, leptin treatment inhibited 5% of cells, consistent with an NPY phenotype.

**FIGURE 2:**
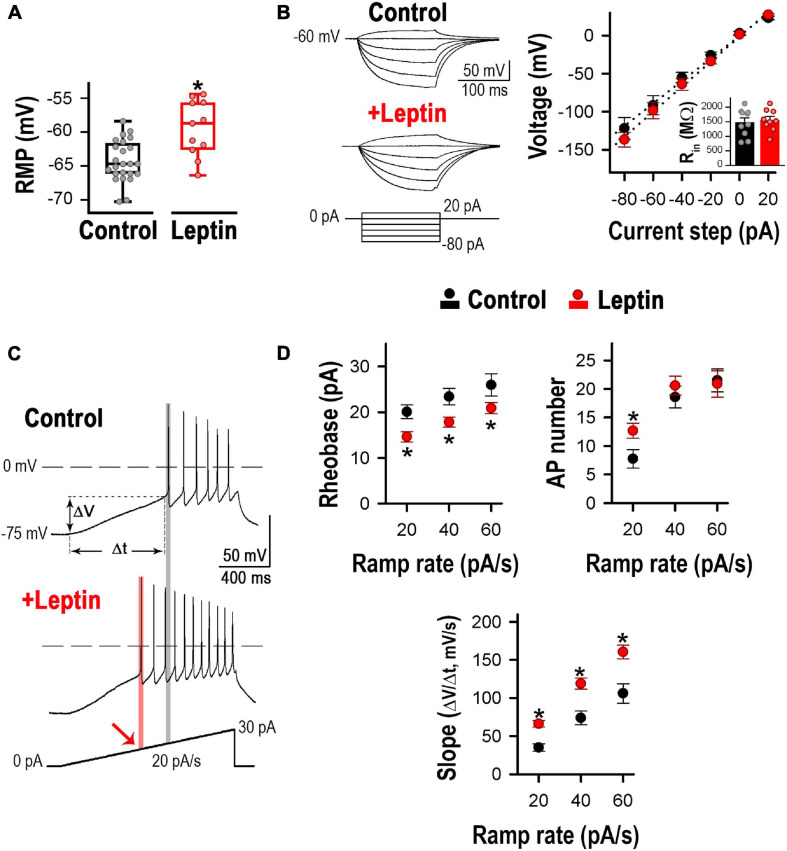
Leptin increases excitability in cultured hypothalamic neurons. **(A)** Leptin application induced a ∼6 mV depolarization. Resting membrane potential (RMP) from control (*n* = 24) and leptin-treated (*n* = 11) neurons. *Significantly different from control, *P* < 0.05, *t*-test (*t* = 3.923, df = 33). **(B)** Input resistance (R_*in*_) from control (*n* = 10) and leptin-treated neurons (*n* = 9). Representative voltage traces (left) and average voltage-current (V-I) relation (right) in response to 200 ms current steps from –80 to 20 pA (every 20 pA, preset membrane potential = –60 mV). R_*in*_ was calculated as the slope of the linear regression. No changes in R_*in*_ were observed between control and leptin treatments (*P* > 0.05, *t*-test, *t* = 0.3883, df = 17). **(C)** Examples of neuronal firing in control and leptin-treated neurons elicited from a preset membrane potential of –75 mV by a 20 pA/s ramp. The rheobase is indicated with the gray line in control and the pink line and arrow in leptin-treated neurons. **(D)** Quantification of rheobase (pA), number of action potentials, and rate of membrane potential depolarization (Slope = ΔV/Δt, mV/s, see panel **C**) in control (*n* = 24, 24, 12) and leptin-treated (100 nM, *n* = 19, 19, 15) neurons in response to 20, 40 and 60 pA/s ramps. *Significantly different from control, *P* < 0.05, *t*-test. Rheobase: *t* = 2.744 for 20 pA/s, *t* = 2.565 for 40 pA/s, *t* = 1.718 for 60 pA/s, df = 41. AP number: *t* = 2.257 for 20 pA/s, *t* = 0.766 for 40 pA/s, *t* = 0.198, df = 41. Slope: *t* = 4.030 for 20 pA/s, *t* = 2.908 for 40 pA/s, *t* = 2.894 for 60 pA/s, df = 25.

The properties of leptin-activated neurons were consistent with POMC neuron responses. As seen in Figure 2A, their resting membrane potential (RMP) was slightly depolarized, whereas their input resistance remained unaltered upon application of 100 nM leptin (Figure 2B). We assessed the effect of leptin on excitability. Membrane excitability was quantified using depolarizing current ramps (20, 40, and 60 pA/s) from a preset membrane potential of ∼−75 mV to avoid spontaneous tonic firing. To analyze quantitatively the voltage response to the current ramps, three different measurements were calculated: the rheobase, the number of APs triggered, and the rate of membrane potential depolarization preceding the first AP (i.e., the slope of depolarization, ΔV/Δt). Figure 2C shows an example of neuronal firing evoked in a POMC neuron at ramp rates of 20 pA/s. Leptin treatment on its own decreased the rheobase from 18.4 to 14.1 pA, increased the number of action potentials from 6 to 11 and enhanced the rate of membrane potential depolarization from 30.0 to 65.0 mV/s; according to the leptin-mediated depolarization of the RMP that was observed in Figure 2A. The summary of effects of leptin on neurons is depicted in Figure 2D. On average, for all tested current ramp rates, leptin decreased the rheobase by ∼25% and increased the rate of membrane potential depolarization between ∼60 and 100%. However, leptin increased the AP number by ∼64% only at ramp rates of 20 pA/s.

### T-Type Calcium Currents Are Necessary for the Leptin-Induced Excitability Response

Due to their ability to conduct calcium across the cellular membrane at potentials close to the resting potential, T–type calcium channels are critically important for regulating neuronal excitability (Llinás, 1988; Huguenard, 1996; Perez-Reyes, 2003). Therefore, we were interested in studying the role of T-type calcium currents in the leptin-induced excitability response.

**FIGURE 3:**
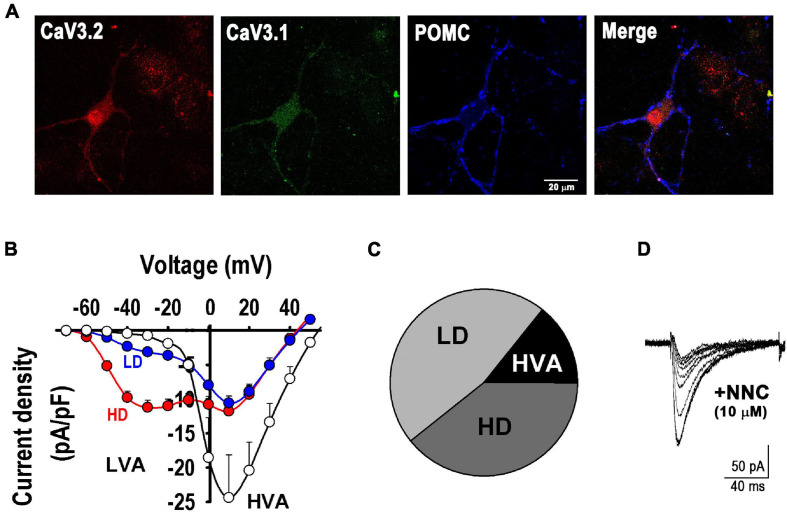
T-type calcium channels in POMC neurons. **(A)** Example of fluorescence images showing the expression of T-type α_1H_ (Ca_*V*_3.2, red) and α_1G_ (Ca_*V*_3.1, green) channels in a POMC neuron (blue). Bar size, 20 μm. **(B)** I-V curves depict three distinct neuron groups (*n* = 28): empty circles, neurons expressing only high-voltage activated (HVA) currents; blue circles, neurons expressing low HVA and low LVA current density levels (LD, I_*LVA peak*_ < 6 -pA/pF); and red circles, neurons expressing low HVA and high LVA current density (HD, I_*LVA peak*_ from 6 to 20 -pA/pF). **(C)** Pie chart denotes the percentual number of neurons in each group described in **(A)**. **(D)** T-type calcium current blockade by its specific blocker NNC 55-0396 (10 μM); the trace amplitude decrement sequence is shown every 2 min before (larger trace) and during application of the drug.

First, we confirmed the presence of Ca_*V*_3.1 and Ca_*V*_3.2 channels in POMC^+^ neurons by immunocytochemistry (Figure 3A) and characterized the calcium currents present in our cultures, including T-type currents (low-voltage gated, LVA). Analysis of Ca^2+^ currents properties revealed three distinct neuron groups, as seen in the I-V curves depicted in Figure 3B. Of all neurons sampled (*n* = 28), 14.3% expressed only high-voltage activated (HVA) currents; 46.4% of neurons expressed both HVA and LVA currents at low levels (LD, I_*LVA peak*_ < 6 -pA/pF), and 39.3% expressed low HVA and high LVA current density levels (HD, I_*LVA peak*_ from 6 to 20 -pA/pF) (Figures 3B,C). Currents elicited by a depolarizing pulse from −90 to −30 mV were confirmed to be T-type by their sensitivity to their specific channel blocker NNC 55-0396 (10 μM, Figure 3D).

**FIGURE 4:**
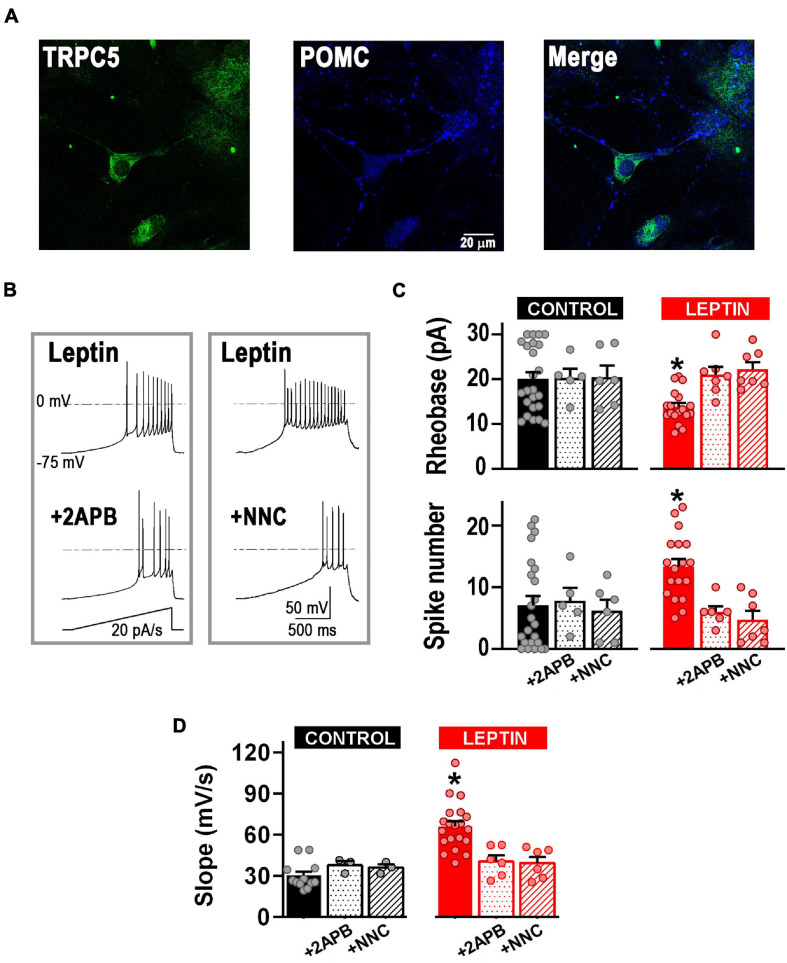
T-type calcium channels are necessary for the leptin-induced excitability response. **(A)** Example of fluorescence images showing the expression of TRPC5 channels (green) in POMC neurons (blue). **(B)** Examples of neuronal firing elicited from a preset membrane potential of –75 mV by a 20 pA/s ramp in leptin-treated neurons before and after the addition of the TRPC channel blocker 2-APB (100 μM) or the T-type channel blocker NNC 55-0396 (10 μM). **(C)** Quantification of rheobase (pA) and number of action potentials in control neurons (*n* = 24), neurons treated with the TRPC channel blocker 2-APB (100 μM, *n* = 5), neurons treated with the T-type channel blocker NNC 55–0396 (10 μM, *n* = 6), neurons treated with leptin (100 nM, n = 18), neurons treated with leptin and 2-APB (n = 5), and neurons treated with leptin and NNC 55-0396 (10 μM, *n* = 7). *Significantly different from all others, *P* < 0.05, ANOVA, *F*_(__5_, _61__)_ = 3.786 (for rheobase), *F*_(__5_, _61__)_ = 3.856 (for AP number). **(D)** Quantification of the rate of membrane potential depolarization (slope) in control neurons (*n* = 12), neurons treated with the TRPC channel blocker 2-APB (100 μM, *n* = 3), neurons treated with the T-type channel blocker NNC 55-0396 (10 μM, *n* = 3), neurons treated with leptin (100 nM, *n* = 20), neurons treated with leptin and 2-APB (*n* = 6), and neurons treated with leptin and NNC 55-0396 (10 μM, *n* = 6). *Significantly different from all others, *P* < 0.05, ANOVA, *F*_(__5_, _44__)_ = 12.77.

We next assessed whether T-type channels are part of the cellular pathway of neurons depolarized by leptin. First, we confirmed the presence of TRPC channels in POMC^+^ neurons in our cultures using immunocytochemistry and western blot analysis (Figures 4A, 6). Membrane excitability in control and leptin-treated neurons was studied before and after the application of the TRPC channel blocker 2-APB (100 μM) or NNC-55-0396 (10 μM) using a ramp rate of 20 pA/s. Figure 4B shows representative examples of leptin-treated neurons before and after aforesaid treatments. In control neurons, neither the application of 2-APB nor the addition of NNC-55-0396 affected neuronal membrane excitability *per se* (Figure 4C, control, *P* > 0.05, ANOVA). However, in leptin-treated neurons, the effect of leptin was abolished after the addition of 2-APB, resulting in a 24.5 ± 4.3% increase in the rheobase and 48.0 ± 5.3% decrease in the number of spikes, compared to the leptin-treated group, corroborating the role of TRPC channels in the leptin-signaling cascade (Figure 4C). Interestingly, the effect of leptin was also prevented solely by the application of the T-type channel blocker NNC-55-0396 (Figure 4C, *P* < 0.05, ANOVA). Similarly, both 2-APB and NNC-55-0396 prevented leptin-induced increase of the rate of membrane potential depolarization (Figure 4D, *P* < 0.05, ANOVA).

**FIGURE 5:**
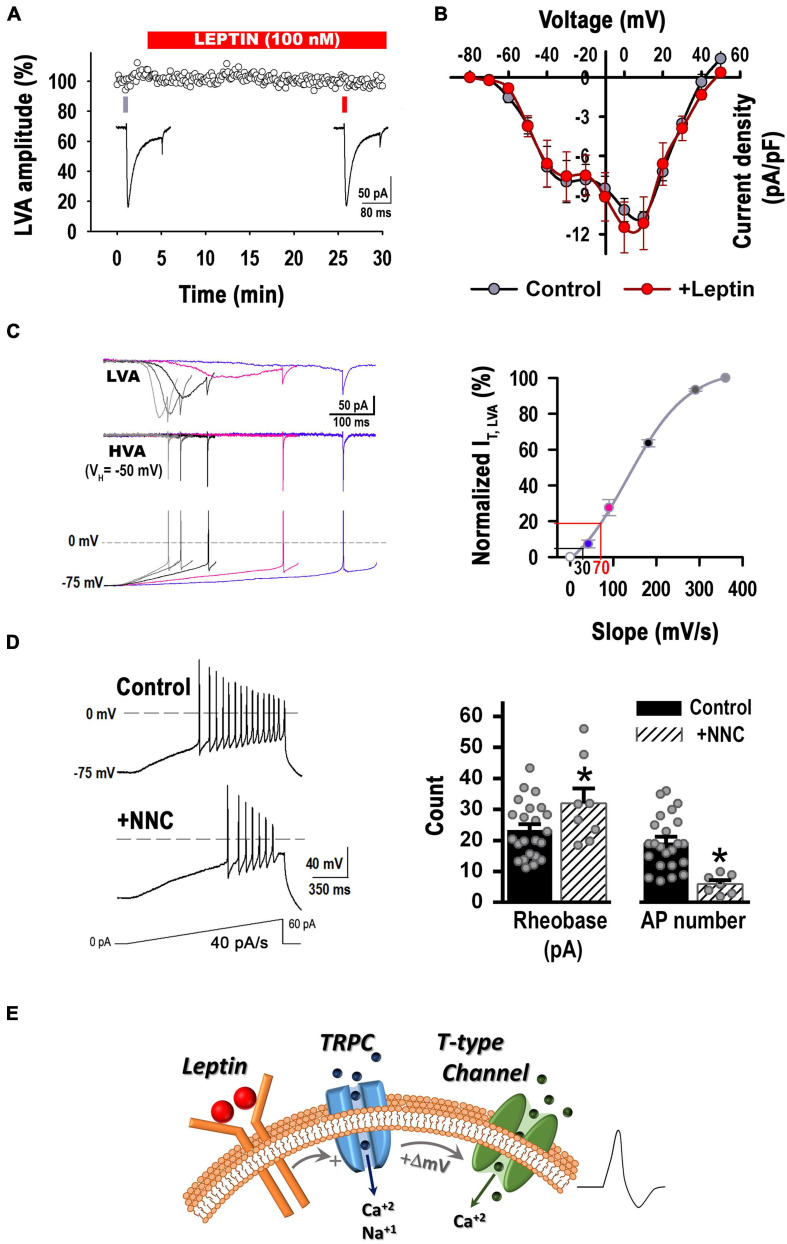
Leptin has no direct effect on T-type current density but increases the membrane depolarization rate favoring T-type channels recruitment. **(A)** Acute incubation with Lep (100 nM) does not alter T-type currents (i.e., LVA currents) elicited by a step voltage from –90 to –30 mV (*n* = 3). **(B)** I-V curves before and after the application of 100 nM leptin in the recording chamber (*n* = 8, paired experiments). Currents were elicited by depolarizing voltage steps (ΔV = 10 mV) from a V_*H*_ of –90 to 50 mV. There is not a statistically significant difference before and after the addition of leptin [*P* > 0.05, Two Way RM ANOVA, *F*_(__1_, _55__)_ = 0.221]. There is not a statistically significant interaction between the voltage and the treatment [*P* > 0.05, Two Way RM ANOVA, *F*_(__11_, _55__)_ = 0.785]. I-V curves from neurons that expressed LVA current at low and high levels were pooled. **(C) Left:** Average traces of T-type currents elicited by various rates of voltage depolarization preceding the first AP. Stimulation protocols (bottom traces) were obtained from hypothalamic neurons recorded in current clamp configuration during current ramp stimulations (20–100 pA/s), which resulted in the following rates of membrane potential depolarization prior the first AP: 40, 90, 180, 290 or 360 mV/s (V_*H*_ = –75 mV). Before stimulation, the membrane potential was held at –90 or –50 mV (V_*H*_) in order to isolate T-type currents. HVA currents recorded from a V_*H*_ of –50 mV (middle traces) were subtracted from LVA and HVA currents recorded from a V_*H*_ of –90 mV, obtaining the isolated LVA component (top traces). **Right:** Normalized T-type current as function of the membrane depolarization rate (slope) (*n* = 5). The black line marks the percentage of non-inactivated T-type current recruited by a depolarizing rate of 30 mV/s. The red line marks the percentage of non-inactivated T-type current recruited by a depolarizing rate of 70 mV/s. **(D) Left:** Example of neuronal firing elicited from a preset membrane potential of –75 mV by a 40 pA/s ramp in a control neuron before and after the addition of the T-type channel blocker NNC 55-0396 (10 μM). **Right:** Application of NNC 55-0396 (*n* = 8) reduced membrane excitability in comparison to control (*n* = 23) (–75 mV, 40 pA/s ramp). *Significantly different from control, *P* < 0.05, *t*-test. Rheobase: *t* = 2.060, df = 29. AP number: *t* = 3.956, df = 29. **(E)** Updated model of leptin’s cellular signaling. Leptin binding to its receptor results in activation of the PI3 kinase signaling pathway and subsequent activation of TRPC channel. TRPC channel activity induces a small but rapid membrane depolarization that recruits T-type channel activity, increasing hypothalamic neuron excitability.

Given that leptin is known to exert direct effects on voltage-gated calcium currents (Takekoshi et al., 2001; Wang et al., 2008), we assessed whether acute incubation with leptin altered T-type LVA currents. As seen in Figure 5A, application of 100 nM leptin in the recording chamber for 30 min did not alter the T-type peak current density (*P* > 0.05, *t*-test, at V_*T*_ = −30 mV). Furthermore, leptin did not affect either LVA or HVA current-voltage relationships (I-V curve, *P* > 0.05, Two Way RM ANOVA, Figure 5B) or the T-type properties of activation or inactivation (not shown), suggesting that the leptin-mediated effect on T-type channels is indirect, likely downstream of TRPC.

So far, our results suggest that T-type calcium currents are as necessary for the leptin-induced excitability response as TRPCs channels. Despite having no direct effect on T-type current density, leptin induced a faster rate of membrane potential depolarization compared to control (Figure 2D); which was prevented after blocking TRPC or T-type channels (Figure 4D). Faster rates of membrane depolarization induce less T-type current inactivation prior to activation of an action potential (Gutierrez et al., 2001), in fact, the results from Figures 4C,D suggest the membrane depolarization rate induced by leptin in response to current ramps of 20 pA/s favors T-type channel recruitment; in contrast, control neurons exhibited slower membrane potential depolarization rates (30.3 ± 2.7 vs. 66.1 ± 4.2 mV/s). The latter conditions favor T-type current inactivation, rendering an inadequate number of available T channels to contribute to the excitability process. Note that NNC-55-0396 did not affect the excitability in control neurons (Figures 4C,D). Therefore, we hypothesize that increased T-type channel availability resulting from the faster leptin-induced membrane potential depolarization rate is sufficient to increase membrane excitability and trigger sodium-mediated action potentials in POMC neurons.

In order to evaluate our hypothesis, we first studied whether T-type channels could be recruited by rates of membrane potential depolarization faster than 30 mV/s in control neurons. We assessed the T-type current activation in voltage-clamp configuration in response to increasing rates of membrane potential depolarization preceding the first AP (left panel, Figure 5C). Peak currents for each rate of membrane potential depolarization were normalized to the maximally elicited current. Average values were plotted against its respective rate of membrane potential depolarization (slope) and fitted using a Boltzmann equation (right panel, Figure 5C). The approximate rate of membrane potential depolarization in response to a ramp rate of 20 pA/s for control (∼30 mV/s, black lines) and leptin-treated (∼70 mV/s, red lines) neurons are shown on the voltage axis of Figure 5C. The lines interpolating the *Y*-axis denote the percentage of non-inactivated I_*T*_ that is recruited in response to the respective rate of membrane potential depolarization. In control neurons (black line), only around ∼5% of I_*T*_ is available for recruitment by a membrane depolarization rate of ∼30 mV/s. However, membrane depolarization rates similar to those induced by leptin-treatment (∼70 mV/s) would be able to recruit 20% of I_*T*_. Thus, control neurons were stimulated in current-clamp configuration with depolarizing current ramps of 40 pA/s, which produce on average a membrane depolarization rate of 73.8 ± 9.1 mV/s. As seen in Figure 5D, NNC-55-0396 not only caused a large increase in the rheobase, but also significantly decreased the number of AP in control neurons. These results confirm that fast rates of membrane depolarization in response to excitatory stimuli recruit enough non-inactivated T-type channels to contribute to the membrane excitability.

Overall, our results show that T-type channel recruitment is necessary in leptin-mediated pathway, likely downstream of TRPC channel activity, as summarized in the cartoon in Figure 5E.

### T-type Calcium and TRPC1/5 Channels Are Expressed in a Functional Complex

**FIGURE 6:**
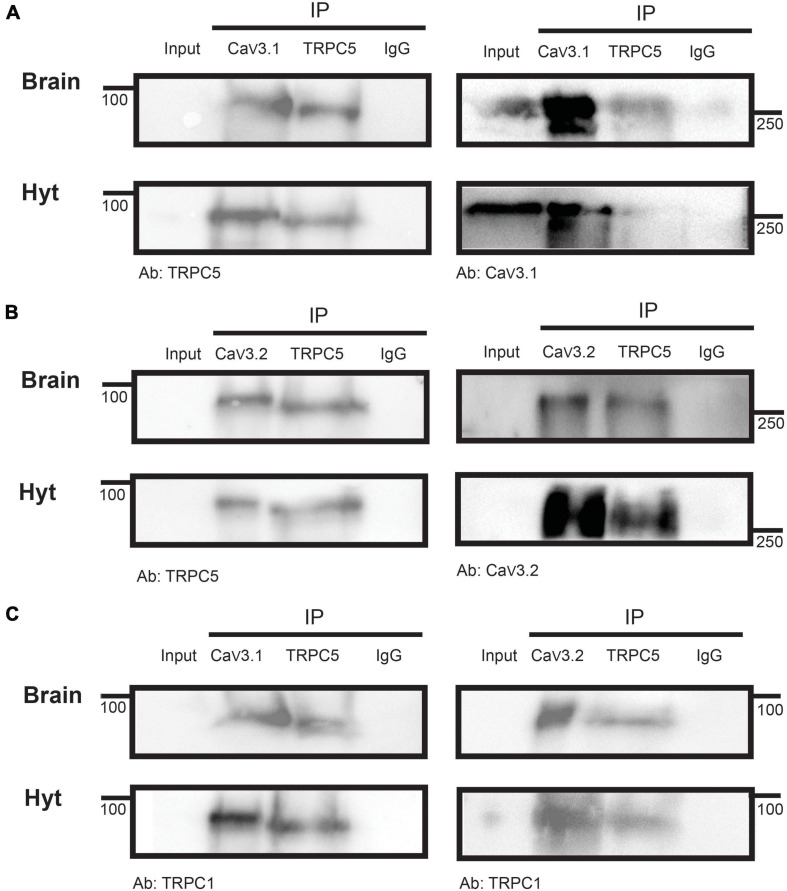
Ca_*V*_3 co-precipitation with TRPC5. Immunoprecipitation of brain and hypothalamus (Hyt) extracts with Ca_*V*_3.1 **(A)** or Ca_*V*_3.2 **(B)** and TRPC5 in the same samples show their mutual interaction. **(C)** IP of brain and Hyt samples using Ca_*V*_3.1 (left) or Ca_*V*_3.2 (right) and TRPC5 show their interaction with TRPC1. IgG was used as IP negative control in all cases, (*n* = 3 for all panels).

We explored whether TRPC and T-type channels exist in a complex. TRPC1/5 complexes are likely the physiological mediators of leptin’s effects in the hypothalamus (Strübing et al., 2001), therefore we assessed whether Ca_*V*_3 channels co-exist with TRPC5 channels in mouse whole brain and hypothalamus samples. Figure 6A shows that Ca_*V*_3.1 channels co-precipitate with TRPC5 channels (left), and similarly, TRPC5 channels co-precipitate with Ca_*V*_3.1 channels (right panel). Ca_*V*_3.2 channels also co-precipitate with TRPC5 channels (Figure 6B, left), and TRPC5 interact with Ca_*V*_3.2 (right). We also determined that TRPC1 is detected in samples co-precipitated with TRPC5 and Ca_*V*_3.1 (left) or Ca_*V*_3.2 (right) (Figure 6C), confirming a TRPC multimer formed by TRPC1/5 is present in the hypothalamus.

**FIGURE 7:**
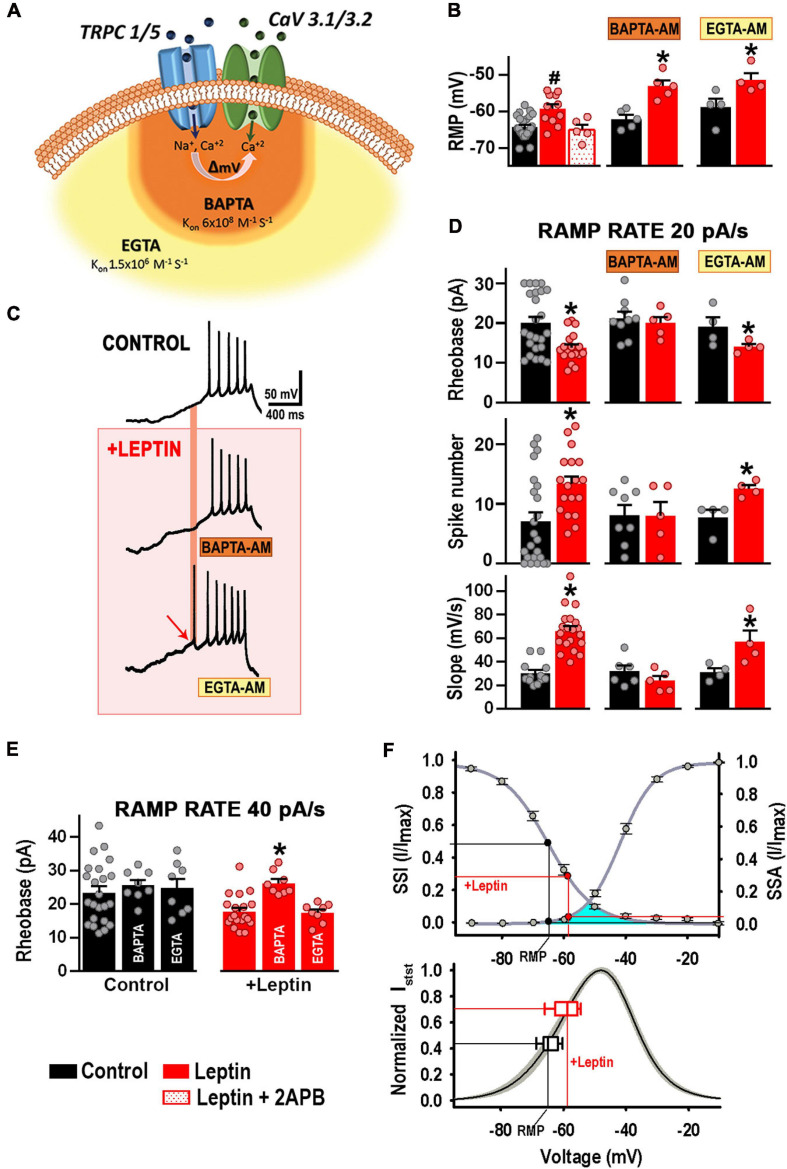
Ca^2+^ influx via TRPC1/5 depolarizes and recruits T-type channels. **(A)** Cartoon depicting T-type calcium channels co-exist in a complex with TRPC1/5. Membrane depolarization induced by TRPC channel-mediated cation influx recruits T-type channel activity. Chelation of Ca^2+^ influx with a fast chelator that binds Ca^2+^ closer to the mouth’s pore (BAPTA-AM, K_*ON*_ = 6 × 10^8^) is represented in orange. Chelation of Ca^2+^ influx with a slower chelator (EGTA-AM, K_*ON*_ = 1.5 × 10^6^) is represented in light orange. **(B)** Resting membrane potential (RMP) from control (*n* = 24) and leptin-treated neurons in the absence (*n* = 11) or presence of 2APB (*n* = 5). Values for control and leptin-treated neurons were reproduced from **(A)** for comparison. Cultured hypothalamic neurons were incubated with either 10 μM BAPTA-AM (*n* = 5) or EGTA-AM (*n* = 4) for 30 min at 37°C prior to whole-cell current clamp to assess leptin-mediated effects on the RMP. ^#^Significantly different from others, *P* < 0.05, ANOVA, *F*_(__2_, _37__)_ = 8.927. *Significantly different from control, *P* < 0.05, *t*-test. BAPTA: *t* = 4.743, df = 8. EGTA: *t* = 2.579, df = 6. **(C)** Example traces of APs elicited by a 20 pA/s ramp protocol under control conditions (top trace), and after the addition of 100 nM leptin in the presence of BAPTA (middle) or EGTA (bottom). **(D)** Rheobase, AP number, and slope values elicited by 20 pA/s ramps for each treatment (*n* = 4–24). *Significantly different from control, *P* < 0.05, *t*-test. Rheobase: *t* = 3.285 (df = 40, control), *t* = 0.4757 (df = 12, BAPTA incubation), *t* = 2.019 (df = 6, EGTA incubation). AP number: *t* = 3.065 (df = 39, control), *t* = 0.044 (df = 11, BAPTA), *t* = 3.376 (df = 6, EGTA). Slope: *t* = 6.495 (df = 30, control), *t* = 1.245 (df = 9, BAPTA incubation), *t* = 2.446 (df = 6, EGTA incubation). **(E)** Rheobase values elicited by 40 pA/s ramps for each treatment (*n* = 8–23). *Significantly different from others, *P* < 0.05, ANOVA, *F*_(__2_, _36__)_ = 0.2637 for control neurons, *F*_(__2_, _32__)_ = 12.36 for leptin-treated neurons. **(F)** T-type channel availability (SSI) and open probability (SSA) as function of voltage. SSI parameters: V_50_ = –65.4 ± 1.0 mV, k = 7.1 ± 0.5 (*n* = 21). SSA parameters: V_50_ = –41.8 ± 0.7 mV, k = 5.2 ± 0.3 (*n* = 22). Black circles indicate the availability and the open probability of T-type channels) at the indicated RMP. Red circles indicate steady sate properties at depolarized RMP after leptin (100 nM) incubation. Window current, underneath SSA and SSI curves is indicated in turquoise. Window currents were calculated for each recording neuron from the SSA and SSI curves fitted with the Boltzmann function (*n* = 18, see section “Materials and Methods”); SEM is indicated in gray. The steady-state current was normalized to its maximum value (Normalized Isst). Steady-state current at the RMP (empty black box plot) increased after incubation with leptin (empty red box plot).

Because T-type calcium channels co-exist in a complex with TRPC1/5, it is possible that local membrane depolarization induced by TRPC1/5 channel-mediated cation influx recruits T-type channel activity. As the ionic selectivity for TRPC1/5 channel complexes is almost 1:1 for Na^+^ and Ca^2+^, Ca^2+^ ions contribute two thirds of the total TRPC1/5-mediated depolarization. Accordingly, chelation of Ca^2+^ influx with a fast chelator that binds Ca^2+^ closer to the mouth’s pore (BAPTA-AM, K_*ON*_ = 6 × 10^8^) compared to a slower, global chelator (EGTA-AM, K_*ON*_ = 1.5 × 10^6^) should allow us to discern whether the TRPC1/5 and T-type channels complex function within a microdomain (Figure 7A). We incubated cultured hypothalamic neurons with either 10 μM BAPTA-AM or EGTA-AM for 30 min at 37°C prior to whole-cell current clamp to assess excitability. As expected, leptin depolarized the RMP *via* activation of TRPC channels (Figure 7B, *P* < 0.05, ANOVA; RMP values for control and leptin-treated neurons have been reproduced from Figure 2A for comparison). The depolarizing effect of leptin occurred regardless of incubation with either BAPTA or EGTA (Figure 7B, *P* < 0.05, *t*-test), suggesting that the chelation of calcium does not affect the TRPC1/5 function *per se*. Figure 7C shows example traces of APs elicited with a 20 pA/s ramp protocol under control conditions (top trace), and after the addition of 100 nM leptin in the presence of BAPTA (middle) or EGTA (bottom). As expected, the presence of intracellular BAPTA or EGTA did not alter the baseline excitability response compared to control (*P* > 0.05, ANOVA; the rheobase and AP number values for control and leptin-treated neurons have been reproduced in Figure 7D from Figure 4 for comparison). Addition of 100 nM leptin in the presence of EGTA caused the expected increase in excitability (*P* < 0.05, *t*-test), in contrast with leptin in the presence of internal BAPTA, which did not elicit significant changes in either the number of APs, rheobase or rate of membrane potential depolarization (*P* > 0.05, *t*-test). Our results suggest that calcium mediates leptin effects on membrane excitability in the proximity of the leptin effector, supporting the idea that TRPC1/5 and T-type channels complex could function within a microdomain.

To further confirm our results, T-type channels were recruited by using depolarizing ramps of 40 pA/s. Neither BAPTA nor EGTA affected membrane excitability in control neurons under those conditions, suggesting no direct effects on the contribution of T-type channels to basal neuronal excitability (Figure 7E). However, only BAPTA prevented leptin-mediated effects on membrane excitability (Figure 7E) in a similar manner as observed with 20 pA/s depolarizing ramps (Figure 7D). These results indicate that the BAPTA effects on membrane excitability were restricted to local leptin-mediated recruitment of T-type channels. The lack of effect of EGTA on membrane excitability confirms that leptin does not depend on a global calcium-dependent mechanism to regulate excitability.

Overall, these results support the hypothesis that Ca^2+^ influx via TRPC1/5 and subsequent depolarization enhances the recruitment of Ca_*V*_3 channels, favoring the generation of APs.

We further studied the physiological relevance of this TRPC1/5-CaV3 complex. As seen in Figures 2A, 7B, the application of leptin induced a ∼6 mV depolarization of RMP. Figure 7F (top panel) shows the steady-state activation (SSA) and steady-state inactivation (SSI) properties of T-type current in cultured hypothalamic neurons. The superimposed lines on the graph show the interpolated values of RMP (*Y*-axis) for control (black lines) and leptin-treated neurons (red lines) pinpointing the non-inactivated (available, SSI) and the activable [open probability (PO), SSA] fractions of T-type channels at the respective resting membrane potentials. We calculated the T-type window current using the fitting parameters of the steady-state activation and inactivation curves (see section “Material and Methods”) (Bijlenga et al., 2000; Lambert et al., 2014; Rivero-Echeto et al., 2020), which is shown in turquoise encompassing the region underneath the steady-state curves (Figure 7F, top panel). Of note, T-type channels are hardly functional during control conditions, when the 50% of available channels are not highly activable.

Our results show that the leptin-mediated RMP depolarization induces changes in T-type channel availability (SSI) and open probability (SSA) (Figure 7F, top panel, red line), leading to a voltage right-shift into the active T-type window current range that results in an increase of the steady state T-type calcium current from 40 to 70%, as better seen in the normalized steady state current graph (Figure 7F, bottom panel). These leptin-mediated changes on T-type calcium steady state current ultimately alter the intrinsic excitability of POMC neurons.

## Discussion

Neuronal cultures with fully developed synapses (after 7 DIV) are useful for mechanistic studies, as they display an “adult” pattern behavior, including ion channel expression (Schlick et al., 2010). Here we corroborated that our primary POMC^+^ hypothalamic neurons responded to leptin as expected according to the bibliography. It is well-established that leptin’s effect on POMC neurons is mediated by the activation of TRPC channels (Qiu et al., 2010, 2011, 2014), POMC neurons increased in our system their excitability in response to administration of nanomolar levels of leptin and this effect was mediated through the activation of TRPC channels, in line with the literature (Qiu et al., 2010, 2011, 2014). The present work provides additional detail on this mechanism, demonstrating that TRPC and T-type channels coexist in a physical and functional macromolecular complex, whereby, leptin-induced membrane depolarization *via* TRPC channel activity immediately recruits adjacent T-type channels, which further depolarize POMC neurons triggering an increase in intrinsic excitability.

Hyperpolarization-induced removal of T-type channel inactivation allows for their stimulation by small depolarizations near the resting potential, rendering T-type currents optimal for regulating excitability under physiological conditions near resting state; as such, T-type channels help control neuronal excitability in various hypothalamic nuclei (van den Top et al., 2004; Qiu et al., 2006; Bosch et al., 2009; Zhang et al., 2009). T-type channels have a confirmed role linking thalamocortical central regulation of wakefulness and body weight (Uebele et al., 2009), and they have been proposed as potential therapeutic targets for treating obesity (Chemin et al., 2001; Uebele et al., 2009; Chorvat, 2013). T-type antagonists prescribed for epilepsy, depression, obsessive-compulsive disorder and bulimia nervosa can cause loss of appetite as a side effect (Cookson and Duffett, 1998; Traboulsie et al., 2006; Wilfong and Willmore, 2006). Here we establish T-type channels (Ca_*V*_3) as essential mediators of membrane depolarization and neuronal excitability triggered by activation of the JNK2-PI3K cascade in response to leptin in POMC neurons. Although leptin has many functions, including effects on control of hormone release, immune system, vasculature, development, somatosensory thalamic activity and higher-level cognitive functions (Haynes et al., 1999; Kim and Moustaid-Moussa, 2000; Friedman, 2004; Myers et al., 2008; Domingos et al., 2011; Farr et al., 2014; Perissinotti et al., 2018); one of its prominent roles is as effector of the negative feedback loop, supporting homeostatic control of energy and food intake, and adipose tissue mass (Ahima et al., 1996). The majority of neurons in our hypothalamic cultures were leptin-activated, in line with a higher abundance of POMC positive neurons detected by ICC. In these neurons, leptin induces electrical activity and depolarization *via* binding to LRb, activation of Janus tyrosine kinase 2 and the downstream activation of phosphatidylinositol 3 kinase (PI3K), resulting in activation of transient receptor potential cation channel activity (TRPC) (Harvey, 2007; Qiu et al., 2010, 2011, 2014). Indeed, leptin application to cultured hypothalamic neurons *in vitro* also resulted in membrane depolarization and increased neuronal excitability. Treatment with the TRPC channel blocker 2-APB after leptin application produced a decrease in the excitability, which was measured as an increase in the rheobase and a decrease of both the rate of membrane depolarization and the number of spikes. However, we found that in addition to TRPC channel activation, T-type channel activity is also essential in this pathway. Blockade of T-type channels with NNC 55-0396 completely prevented the effect of leptin, even when TRPC channel activity remained intact. Qiu et al. (2010) demonstrated leptin recruits a non-selective Na^+^ and Ca^2+^ channel sensitive to SKF96365 and APB and potentiated by lanthanum (La^3+^), consistent with a TRPC channel complex. Here, we showed T-type channel function is essential in the response to leptin. The leptin-activated current is strongly potentiated by lanthanum (Qiu et al., 2010), a T-type channel blocker more potent than nickel (Mlinar and Enyeart, 1993). Thus, it’ is unlikely T-type calcium channels are directly activated by leptin, consistent with our data showing leptin does not directly activate these channels and the steady-state properties of these currents. Instead, leptin induced faster rates of membrane depolarization, which in turn produced less T-type current inactivation prior to activation of an action potential (Gutierrez et al., 2001). Therefore, T-type channels are likely recruited downstream of TRPC channel activation by discrete changes in membrane depolarization induced by TRPC channels and are the final mediator of triggered activity. Insulin and leptin engage a common signaling pathway at the cellular level to activate TRPC5/1 channel complexes and depolarize POMC neurons (Qiu et al., 2014). Interestingly, estradiol-mediated upregulation of Cav3.1 channel rendered POMC neurons more excitable and responsive to insulin-mediated TRPC5 channel activation (Qiu et al., 2018).

Furthermore, we demonstrate TRPC1/C5 channels and Ca_*V*_3.1 and Ca_*V*_3.2 channels exist in complex. Calcium channels are known to co-exist with other channels in functional complexes (Robitaille et al., 1993; Vivas et al., 2017), notably T-type channels form protein complexes with members of the potassium channel family such as Kv4, KCa3.1, and KCa1.1 (Anderson et al., 2010; Rehak et al., 2013), which ensures rapid potassium channel activation thanks to their proximity with Ca_*V*_3 channels within the microdomain. Furthermore, TRPC channels are known to assemble in multiprotein complexes that include various key Ca^2+^ signaling proteins within Ca^2+^ signaling microdomains (Ambudkar, 2006). For instance, TRPC1, 3, 4, 5, 6, and 7 isoforms can form a macromolecular complex with the α_1C_ subunit of the L-type voltage-gated calcium channel (Cav1.2) in atria and ventricle of developing heart (Sabourin et al., 2011). Here, co-immunoprecipitation experiments show that TRPC5 interacts with Ca_*V*_3.1 and Ca_*V*_3.2 channels. The functional activity of this complex was corroborated using intracellular calcium chelators; prevention of leptin-induced calcium influx through TRPC channels by intracellular BAPTA (but not EGTA) was sufficient to preclude POMC neuron excitability. Our results agree with those of Qiu et al. (2010), who reported that leptin-induced inward current was reduced by BAPTA but not EGTA. It has been shown that intracellular BAPTA prevents intracellular Ca^2+^-dependent potentiation of the TRPC channel complex *in vitro*, suggesting that calcium binding to an intracellular site in the TRPC channel complex is necessary for its function (Strübing et al., 2001). However, leptin is capable of recruiting a non-selective Na/Ca current in hypothalamic neurons in the presence of BAPTA, albeit smaller than in control conditions but larger than that observed in the absence of extracellular calcium (Qiu et al., 2010). In line with these results, we show that leptin-induced depolarization can occur even in the presence of either BAPTA or EGTA suggesting that the calcium entry thought to be necessary to self-activate the TRPC1/5 complex is not blocked by the presence of BAPTA in hypothalamic neurons.

T-type Ca^2+^ channels operate in a subthreshold voltage range, with an overlap between steady-state activation and inactivation curves that produces a voltage range where a subset of T-type channels are constitutively open (i.e., steady-state current, I_*stst*_ or “window current”) (Hughes et al., 1999; Crunelli et al., 2005). Furthermore, we observed that the leptin-mediated depolarization of RMP induced a voltage right-shift into the active T-type window current range, resulting in an increase of the steady state T-type calcium current from 40 to 70%; which ultimately affects the intrinsic excitability of POMC neurons. This study was focused on the somatic response of POMC cultured neurons from newborn mice, thus it did not address questions pertaining to the hypothalamic circuit level. However, our results show T-type channel activity is necessary for leptin-mediated effects on hypothalamic POMC neuron excitability and confirms T-type channels as possible additional drug targets for leptin-mediated functions, such as metabolic energy regulation and control of food satiety.

## Data Availability Statement

The raw data supporting the conclusions of this article will be made available by the authors, without undue reservation. A previous version of this research is available (https://www.biorxiv.org/content/10.1101/2020.07.21.214296v1).

## Ethics Statement

The animal protocols used in this study were reviewed and approved by an independent Institutional Animal Care and Use Committee at Loyola University Chicago (IACUC 2016032).

## Author Contributions

EP-R did the conception of research, approved the final version of the manuscript, and supervised the experiments. EP-R, EM-H, and PP designed the experiments, performed experiments, analyzed the data, interpreted results of experiments, and prepared the figures. EP-R and PP wrote the manuscript, edited, and revised the manuscript. All authors contributed to the article and approved the submitted version.

## Conflict of Interest

The authors declare that the research was conducted in the absence of any commercial or financial relationships that could be construed as a potential conflict of interest.

## Abbreviations

AgRP: agouti-related peptide
AP: action potential
DIV: days *in vitro*
KLHL1: Kelch-like 1
LRb: Leptin receptor
LVA: low-voltage activated
NPY: neuropeptide Y
PIR: post-inhibitory rebound
POMC: proopiomelanocortin positive neurons
TRPC: Transient Receptor Potential Cation
TRPC: Transient Receptor Potential Cation.
